# A Methodology for Abstracting the Physical Layer of Direct V2X Communications Technologies

**DOI:** 10.3390/s22239330

**Published:** 2022-11-30

**Authors:** Zhuofei Wu, Stefania Bartoletti, Vincent Martinez, Alessandro Bazzi

**Affiliations:** 1 College of Computer Science and Technology, Harbin Engineering University, Harbin 150001, China; 2 WiLab, CNIT/DEI, University of Bologna, 40126 Bologna, Italy; 3 CNIT/DIE, University of Rome Tor Vergata, 00133 Rome, Italy; 4 NXP Semiconductors, 31023 Toulouse, France

**Keywords:** connected vehicles, IEEE 802.11p, C-V2X sidelink, physical layer abstraction, SINR threshold model, implementation loss

## Abstract

Recent advancements in vehicle-to-everything (V2X) communications have greatly increased the flexibility of the physical (PHY) and medium access control (MAC) layers. This increases the complexity when investigating the system from a network perspective to evaluate the performance of the supported applications. Such flexibility, in fact, needs to be taken into account through a cross-layer approach, which might lead to challenging evaluation processes. As an accurate simulation of the signals appears unfeasible, a typical solution is to rely on simple models for incorporating the PHY layer of the supported technologies based on off-line measurements or accurate link-level simulations. Such data are, however, limited to a subset of possible configurations, and extending them to others is costly when not even impossible. The goal of this paper is to develop a new approach for modeling the PHY layer of V2X communications that can be extended to a wide range of configurations without leading to extensive measurement or simulation campaigns at the link layer. In particular, given a scenario and starting from results in terms of the packet error rate (PER) vs. signal-to-interference-plus-noise ratio (SINR) related to a subset of possible configurations, we first approximated the curves with step functions characterized by a given SINR threshold, and we then derived one parameter, called implementation loss, that was used to obtain the SINR threshold and evaluate the network performance under any configuration in the same scenario. The proposed methodology, leading to a good trade-off among the complexity, generality, and accuracy of the performance evaluation process, was validated through extensive simulations with both IEEE 802.11p and LTE-V2X sidelink technologies in various scenarios. The results first show that the curves can be effectively approximated by using an SINR threshold, with a value corresponding to 0.5 PER, and then demonstrate that the network-level outputs derived from the proposed approach are very close to those obtained with complete curves, despite not being restricted to a few possible configurations.

## 1. Introduction

Vehicle-to-everything (V2X) connectivity allows vehicles to communicate with one another and with other road elements to share local views and intentions, discover surroundings, and coordinate driving maneuvers [1,2], improving the safety and efficiency of our transportation systems [3]. Focusing on direct communications, two families of standards have been defined for direct V2X connectivity, i.e., the one based on IEEE 802.11p, which is expected to be shortly amended by the IEEE 802.11bd, and the other based on the sidelink technologies designed by the 3GPP for V2X, which today means long term evolution (LTE) and 5G new radio (NR), and might become 6G in the next decade.

The new developments in V2X standardization have enabled greater flexibility at both the physical (PHY) and medium access control (MAC) layers, with the scope to enable a large variety of use cases. This calls for a cross-layer performance analysis, where the main PHY and MAC layer parameters and procedures, as well as the interplay between them, should be considered to cover a variety of different scenarios and settings. One of the main issues when investigating V2X over multiple layers is how to reproduce the performance of the PHY layer in a sufficiently accurate way without overly impacting the computational complexity. An accurate simulation of the PHY layer would in fact require, in principle, a bit-by-bit generation per transmitter and every receiver, a conversion to electromagnetic signals, and propagation through a multi-path variable channel. However, introducing an accurate signal-level simulation of the PHY layer makes network-level simulations very slow and, in some cases, unfeasible, due to the large vehicle densities and different levels of mobility that need to be investigated. Additionally, it is worth noting that V2X transmissions are normally in broadcast mode, leading to a very high number of links to be evaluated.

Instead of performing detailed PHY layer simulations, a commonly adopted approach is to use packet error rate (PER) vs. (average) signal-to-interference-plus-noise ratio (SINR) curves, such as those presented in [4]. More specifically, per each message exchange, the SINR is calculated based on the position of the nodes, including path-loss, small-scale fading, and the interference received from other signals, and then the correctness is statistically determined based on the given curve. This approach is supported by the fact that, within V2X communications, the channel normally varies quickly enough that the small-scale fading observed by different transmissions in the time domain can be assumed as uncorrelated [5]. Despite this being a widely adopted solution, the main problem is that different SINR vs. PER curves have to be generated for each scenario or system setting, i.e., any technology, packet size, modulation and coding scheme (MCS), and link-level simulations or on-field measurements required for generating these curves are usually computationally intensive or operationally unfeasible. It is worth noting that there exist a plethora of configurations for V2X communications, as the packet size is variable [6] and the MCS might be adapted to channel conditions [7,8].

In this work, we provide a methodology for abstracting the PHY layer in network-level simulations that, starting from a few available PER vs. SINR curves, allows us to extend to a large set of configurations without impacting the accuracy of the results.

### 1.1. Related Work

The most accurate approach when simulating wireless networks is to reproduce all of the processes from bits to signals, propagation, and reception with decoding. However, this comes at a very high cost in terms of memory and time, and it is in fact unfeasible when a large number of nodes is considered. This is emphasized in V2X due to the broadcast nature of transmissions, which implies that several signals and decoding attempts need to be evaluated per each transmission. As an example, in [9], the authors propose a complete V2X simulator with an accurate PHY layer, which is shown to behave differently to another simulator, where the PHY layer is simplified. However, the difference appears limited (less than 1 dB looking at the packet success rate vs. signal-to-noise ratio (SNR)) and the time required to simulate 30 s goes from 1000 s with 15 vehicles to more than 5000 s with 30 vehicles, suggesting that simulating hundreds of vehicles with this approach is not needed and almost unfeasible.

The most common approach is therefore to use PER vs. SINR curves as already discussed. A few examples of works where these kinds of curves are used are [10,11], where LTE-V2X is studied, or [12], where the subject is IEEE 802.11p. Many research activities have therefore been devoted in the last decade to assessing the performance of direct V2X communications standards and providing PER vs. SINR as an output of their work. Just as a few examples, curves are reported for IEEE 802.11p in [13] through measurements and in [14] through accurate link-level simulations, curves for the LTE-V2X sidelink are shown in [4], also comparing the impact of different demodulation reference signal (DMRS) configurations, and curves comparing IEEE 802.11p with the LTE-V2X sidelink are provided in [15], with a particular emphasis on the frequency offset estimation. In some cases, not only are some PER vs. SINR curves provided, but they are also used to assess the performance at the network level. This is the case for the example of [16,17], where IEEE 802.11p and the LTE-V2X sidelink are compared covering some MCSs.

Given that curves are provided for a limited set of configurations, attempts were carried out to implement some methodology for increasing the validity of the link-level simulations when used to abstract the PHY layer in network-level simulations. In [18,19] in particular, the SINR is calculated on a subcarrier basis and then converted to an overall effective SINR to be used in PER vs. SINR curves derived in the additive white Gaussian noise (AWGN) channel. The proposal, despite being able to cover more situations and being applicable to both the cellular and IEEE technologies, relies on one parameter that needs to be obtained per each configuration via detailed link-level simulations, eventually failing to provide a way to generalize parameter settings that were not considered.

In parallel to this, a number of works further simplify the PHY layer modeling by using a single SINR threshold instead of the PER vs. SINR curve, which clearly further reduces the burden of simulating the transmission–reception process and also simplifies the modeling in analytical studies. For example, an SINR threshold-based model is used to investigate LTE-V2X in [20] and for the validation of new proposals in [21], addressing a new MAC protocol based on network coding, and, in [22], focusing on software-defined networking. The problem with this approach is that the value of the threshold is often either selected arbitrarily, as in [22], or starting from a PER vs. SINR curve but without justification, as in [20], where the SINR value corresponding to 0.01 PER is adopted, or in [21], where the value of the SINR corresponding to the lowest PER is used. An approach used to derive such a threshold from PER vs. SINR curves and a demonstration that the use of thresholds can be an acceptable approximation is still missing.

Summarising, the approaches used in the literature to abstract the PHY layer in network-level simulations either become unfeasible when a large number of vehicles is assumed or appear as having limited flexibility when the configuration changes.

### 1.2. Contribution and Innovation of the Paper

In this paper, we propose a methodology for the PHY abstraction of V2X communications, which extends the PHY-level results available for a few configurations; it can be used at the network level for mathematical models or simulations, and, in the latter case, it implies a very reduced impact on the processing and memory consumption. Specifically, we present a methodology used to derive a parametric model, with a single parameter called implementation loss that depends on the operating scenario.

We started from PER vs. SINR curves and approximated them with step functions, i.e., the packet is correctly received if the SINR is above a given threshold, hereafter called the SINR threshold, and discarded if it is below. The approximation is shown to be sufficiently accurate for the most relevant configurations of traffic densities and technologies. Then, we extended the calculation of the SINR threshold to those configurations for which we do not have the SINR vs. PER curve without the need of additional and costly measurements or link-level simulations.

The proposed methodology was validated by using it in network-level simulations. As a benchmark, the same evaluations were also carried out by relying on the PER vs. SINR curves obtained through link-level simulations, which are, in principle, more accurate yet computationally intensive and limited to a few configurations. The results show that the model resulting from the proposed methodology leads to an accurate evaluation at the network level of direct V2X communications technologies, with a negligible impact on the processing speed and being able to cover a high number of relevant cases without the need of additional and heavy campaigns of measurements or link-level simulations.

### 1.3. Paper Organization

The rest of the paper is organized as follows. The PHY layer and MAC layer of IEEE 802.11p and the LTE-V2X sidelink are briefly recalled in Section 2. Section 3 presents the proposed methodology for PHY layer abstraction followed by the validation in Section 4. Finally, our conclusion is given in Section 5.

## 2. V2X Technologies

The main families of technologies for direct V2X communications are currently those based on IEEE 802.11p and those under the umbrella of cellular-V2X (C-V2X) and denoted as a sidelink. The two families rely on orthogonal frequency-division multiplexing (OFDM) at the PHY layer and differ in the access mechanisms at the MAC layer [23]. In this section, we recall the mechanisms at the MAC layer for both of them and define the transmission time for a generic payload of $P_{b}$ bytes. The duration of the generic transmission is required for the calculation of the effective throughput in Section 3.

### 2.1. IEEE 802.11p

IEEE 802.11p is an approved amendment to the IEEE 802.11 standard for the PHY and MAC layer of vehicular communications. In the PHY layer, IEEE 802.11p operates in the 5.9 GHz ITS band and uses OFDM with a 10 MHz bandwidth. Each OFDM symbol includes 52 subcarriers with a subcarrier spacing of 156.25 kHz (4 of them used as the pilots), and lasts 8 μs. There are eight possible combinations of MCS, with the modulation ranging from BPSK to 64-QAM and the encoding implemented through a convolutional code with rate 1/2, possibly punctured to reach 2/3 or 3/4. The signal transmitted at the PHY layer consists of a preamble field (32 μs), a signal field (8 μs), and a data field (variable time). More details can be found in [24].

The MAC algorithm deployed by IEEE 802.11p is called enhanced distributed coordination access (EDCA). It is based on the basic distributed coordination function (DCF) but adds QoS attributes. DCF is a carrier sense multiple access with collision avoidance (CSMA/CA) algorithm. In CSMA/CA, a node listens to the channel before transmission and, if the channel is perceived as idle for a predetermined time interval, the node starts to transmit. If the channel becomes occupied during such an interval, the node performs a backoff procedure, i.e., the node defers its access according to a randomized time period. In IEEE 802.11p, the predetermined listening period is called arbitration inter-frame space (AIFS) [25]. Therefore, we can calculate the time required to transmit a packet with a given payload $P_{b}$ on the wireless medium as [18]:

(1) $$\begin{array}{c} T_{\mathrm{tx}}^{\left(11p\right)}=T_{\mathrm{AIFS}}+T_{\mathrm{pre}}+T_{\mathrm{sym}}n_{\mathrm{sym}} \end{array}$$

where $T_{\mathrm{AIFS}}$ is the duration of the AIFS, $T_{\mathrm{pre}}$ is the preamble duration (40 μs, including the preamble field and the signal field), $T_{\mathrm{sym}}$ is the OFDM symbol duration (8 μs), $n_{\mathrm{sym}}=\left\lceil 8P_{b}/n_{\mathrm{bpS}}\right\rceil$ denotes the number of OFDM symbols required to transmit a certain payload (including MAC header, service, and tails bits), and $n_{\mathrm{bpS}}$ is the number of data bits per OFDM symbol [26].

### 2.2. C-V2X Sidelink

At the lower layers, sidelink numerology and building blocks of C-V2X are based on the uplink specifications, which are single carrier frequency division multiple access (SC-FDMA) in LTE-V2X and cyclic prefix orthogonal frequency-division multiplexing (CP-OFDM) in 5G-V2X. LTE-V2X operates in 10 MHz or 20 MHz channels, whereas 5G-V2X can occupy up to 100 MHz when used in bands below 6 GHz (namely, sub 6 GHz). The resources are based on a time–frequency matrix structure, where the time domain is divided into a transmission time interval (TTI) of 1 ms duration in LTE-V2X and of either 0.25 ms, 0.5 ms, or 1 ms in 5G-V2X (sub 6 GHz).

In the frequency domain, radio resources are organized in resource elements (REs), which aggregate into physical resource blocks (PRBs), in turn realizing the subchannels. Each RE is a subcarrier (spaced by 15 kHz in LTE and 15, 30, or 60 kHz in 5G) over an OFDM symbol. Each PRB is composed of 12 consecutive subcarriers in the frequency domain with the same subcarrier spacing (SCS). The sub-channels are composed of a certain number of PRBs. As the SCS changes, the bandwidth of a PRB varies accordingly. As a result, the number of PRBs and subchannels within a fixed channel bandwidth depends on the SCS.

A packet is normally transmitted on one or more subchannels within one TTI, which lasts 1 ms in LTE-V2X and either 0.25, 0.5, or 1 ms in 5G-V2X, depending on the SCS. In principle, the transmission can be split over more than one TTI if the packet size and adopted MCS require more subchannels than those that are available. Therefore, we can calculate the time required to transmit a packet as

(2) $$\begin{array}{c} T_{\mathrm{tx}}^{\left(C\text{-}V2X\right)}=T_{\mathrm{TTI}}\left\lceil \frac{n_{\mathrm{PRB}\text{-}\mathrm{pkt}}}{n_{\mathrm{PRB}\text{-}\mathrm{TTI}}}\right\rceil =T_{\mathrm{TTI}}\cdot n_{\mathrm{TTI}} \end{array}$$

where $T_{\mathrm{TTI}}$ is the TTI duration, $n_{\mathrm{PRB}\text{-}\mathrm{pkt}}$ is the number of PRBs necessary for one packet transmission (which depends on $P_{b}$ and the adopted MCS [27]), $n_{\mathrm{PRB}\text{-}\mathrm{TTI}}$ is the number of PRBs in a TTI [28], and $n_{\mathrm{TTI}}=\left\lceil \frac{n_{\mathrm{PRB}\text{-}\mathrm{pkt}}}{n_{\mathrm{PRB}\text{-}\mathrm{TTI}}}\right\rceil$ is the number of TTIs needed for transmitting the packet. In most of the cases, the transmission lasts a single TTI; thus, $n_{\mathrm{TTI}}=1$ and $T_{\mathrm{tx}}^{\left(C\text{-}V2X\right)}=T_{\mathrm{TTI}}$.

## 3. Physical Layer Abstraction Methodology

In this section, we propose a general methodology used to leverage a set of available curves under specific settings to obtain a more general PHY layer abstraction to be used in the network-level simulations of V2X communications. Hereafter, we first briefly describe the main ideas and assumptions, and then provide the details of the methodology through Section 3.1, Section 3.2, Section 3.3, Section 3.4 and Section 3.5.

As a starting point, we approximate the PER vs. SINR curves using step functions, corresponding to a certain SINR threshold $\gamma_{\mathrm{th}}$. With this approximation, if the SINR calculated at the receiver is above the threshold, the decoding is successful; otherwise, the message is lost. Note that, as already discussed in Section 1.1, the use of a step function to approximate the PER vs SINR curve is often performed in literature, especially when analytical models are derived. However, to the best of our knowledge, no detailed studies have been performed to discuss which SINR threshold should be selected given the curve, nor to demonstrate that the approximation is acceptable. Differently, hereafter, in Section 3.1, we detail an approach used to derive the SINR threshold and we show in Section 4.1 that the impact of the deriving approximation is very limited when focusing on network-level simulations.

Given the derivation of the SINR threshold for the few curves that are available, we then propose a way to also infer the SINR threshold for configurations where the curves are not available. The methodology is illustrated in Figure 1 and performed in two steps. Step 1 starts from a given scenario, for which, the SINR threshold can be obtained from available PER vs. SINR curves corresponding to a subset of configurations, i.e., for some technologies, MCSs, and packet sizes (in our work, curves are available for a highway scenario in line-of-sight (LOS) and non-line-of-sight (NLOS) conditions and for an urban scenario in LOS and NLOS conditions, and in all cases for a set of settings of both IEEE 802.11p and the LTE-V2X sidelink). For these configurations, we calculated a parameter, which is called implementation loss and denoted as α. The calculation of α was based on the Shannon–Hartley theorem: we calculated the maximum throughput in an AWGN channel $\Psi_{s}$ for that SINR value (Section 3.2) and the effective throughput $\Psi_{e}$ of the given technology and settings as detailed in Section 3.3; then, we assumed that the effective throughput $\Psi_{e}$ can be approximated as an attenuated form of the maximum throughput $\Psi_{s}$, which is a function of the SINR threshold, i.e.,

(3) $$\begin{array}{c} \Psi_{e}\left(\theta \right)≃\alpha \Psi_{s}\left(\gamma_{\mathrm{th}}\left(\theta \right)\right) \end{array}$$

where α is the implementation loss for that specific scenario and configuration; note that an equation similar to (3) used to approximate the effective throughput starting from a given SINR is often used, an example being [29]. The first step is concluded, as detailed in Section 3.4, by calculating a single value for the implementation loss, denoted as $\hat{\alpha }$, which is the value that best approximates those obtained from the available curves.

In the second step, for any technology and system settings of interest, the value of $\Psi_{e}$ is calculated and then used together with the implementation loss $\hat{\alpha }$ of the first step to derive the SINR threshold ${\hat{\gamma }}_{\mathrm{th}}$, as detailed in Section 3.5.

### 3.1. PER vs. SINR Curve Approximation

Figure 2 illustrates the method for the derivation of $\gamma_{th}$ from the PER vs. SINR curve. The blue solid line is a given PER vs. SINR curve corresponding to a specific scenario and certain system settings. We obtained $\gamma_{th}$ as the SINR value that corresponds on the curve to a certain PER value β (the asterisk). It follows that, instead of the original PER vs. SINR curve, we now have a step function (represented through an orange dashed line in Figure 2). In order to determine which value of β to use, later called $\hat{\beta }$, the mean absolute error (MAE) was used, calculated through the use of network-level simulations. In particular, the MAE was calculated by looking at the packet reception ratio (PRR) (i.e., the percentage of the packets correctly received at a given distance), varying the source-destination distance as:

(4) $$\begin{array}{c} MAE=\frac{1}{n}\cdot \sum_{i=1}^{n}\left|{\mathrm{PRR}}_{i}^{\left(sf\right)}\left(\beta \right)-{\mathrm{PRR}}_{i}^{\left(curve\right)}\right| \end{array}$$

where ${\mathrm{PRR}}_{i}^{\left(sf\right)}\left(\beta \right)$ and ${\mathrm{PRR}}_{i}^{\left(curve\right)}$ are the *i*-th PRR value point in the PRR vs. distance curves (e.g., see Figure 3). By minimizing the MAE, the best value $\hat{\beta }$ is obtained.

### 3.2. Maximum Throughput $\Psi_{s}$

From the SINR threshold $\gamma_{th}$, the channel capacity as defined by the Shannon–Hartley theorem, i.e., the maximum theoretical throughput $\Psi_{s}$ that can be achieved over an AWGN channel for a given SINR, is calculated as

(5) $$\begin{array}{c} \Psi_{s}\left(\gamma_{\mathrm{th}}\right)=B\log_{2}\left(1+\gamma_{\mathrm{th}}\right) \end{array}$$

where *B* is the bandwidth of the channel and $\gamma_{\mathrm{th}}$ is the SINR threshold.

### 3.3. Effective Throughput $\Psi_{e}$

The effective throughput is defined as the maximum net throughput for the given configuration [18]. In particular, given the packet size $P_{b}$ and the MCS, the effective throughput is calculated as the ratio between the number of data bits and the time required for the transmission, which means for IEEE 802.11p and C-V2X that it can be calculated using (1) and (2) as:

(6) $$\begin{array}{cc} \Psi_{e}^{\left(11p\right)}\left(\theta^{\left(11p\right)}\right) & =\frac{8P_{b}}{T_{\mathrm{tx}}^{\left(11p\right)}} \end{array}$$

(7) $$\begin{array}{cc} \Psi_{e}^{\left(C\text{-}V2X\right)}\left(\theta^{\left(C\text{-}V2X\right)}\right) & =\frac{8P_{b}}{T_{\mathrm{tx}}^{\left(C\text{-}V2X\right)}}\cdot \frac{n_{\mathrm{subch}}\cdot n_{\mathrm{PRB}\text{-}\mathrm{subch}}}{n_{\mathrm{PRB}\text{-}\mathrm{pkt}}}\; \end{array}$$

where $\theta^{\left(11p\right)}$ and $\theta^{\left(C\text{-}V2X\right)}$ represent the generic system setting vectors for IEEE 802.11p and C-V2X, respectively, i.e.,:

(8) $$\begin{array}{cc} \theta^{\left(11p\right)} & =[T_{\mathrm{pre}},T_{\mathrm{AIFS}},T_{\mathrm{sym}},n_{\mathrm{sym}}] \end{array}$$

(9) $$\begin{array}{cc} \theta^{\left(C\text{-}V2X\right)} & =[n_{\mathrm{subch}},n_{\mathrm{PRB}\text{-}\mathrm{subch}},T_{\mathrm{TTI}},n_{\mathrm{PRB}\text{-}\mathrm{pkt}}] \end{array}$$

with $n_{\mathrm{subch}}$ being the number of subchannels and $n_{\mathrm{PRB}\text{-}\mathrm{subch}}$ the subchannel size, expressed as the number of PRBs. Please note that the number of PRBs in a TTI can be written as a function of the number of subchannels and PRBs per subchannel as $n_{\mathrm{PRB}\text{-}\mathrm{TTI}}=n_{\mathrm{subch}}\cdot n_{\mathrm{PRB}\text{-}\mathrm{subch}}$.

### 3.4. Best Fit Implementation Loss $\hat{\alpha }$

Per each PER vs. SINR curve, the operations detailed in Section 3.1, Section 3.2 and Section 3.3 can be used to calculate the effective throughput and the SINR threshold, which, in principle, allow us to obtain the implementation loss α using (3). However, in the general case, only the effective throughput can be calculated, and both the SINR threshold and the implementation loss α are unknown. In order to relate the effective throughput with the SINR threshold for any possible configuration, the best fit $\hat{\alpha }$, which best approximates the value of α in the known cases, is derived.

Specifically, assume that there are *N* available PER vs. SINR curves within a specific scenario. Each curve corresponds to specific parameter settings, i.e., $\left\{\theta_{i}|i=1,2,\ldots ,N\right\}$, where $\theta_{i}$ represents a vector that includes the PHY and MAC parameters for the *i*-th settings. In order to estimate the parameter $\hat{\alpha }$, a least-square approach is considered over the set of available curves, i.e.,

(10) $$\begin{array}{c} \hat{\alpha }=\arg\min_{\alpha }\sum_{i=1}^{N}{\left[\Psi_{e}\left(\theta_{i}\right)-\alpha \Psi_{s}\left(\gamma_{\mathrm{th}}\left(\theta_{i}\right)\right)\right]}^{2} \end{array}$$

### 3.5. SINR Threshold for the Generic Settings

Once the value $\hat{\alpha }$ is obtained for the given scenario, as explained in Section 3.4, it can be used for any parameter setting θ beyond those for which a PER vs. SINR curve is available, e.g., for any MCS and for any packet size. The SINR threshold corresponding to the generic θ is in fact obtained by combining (3) and (5) as

(11) $$\begin{array}{c} {\hat{\gamma }}_{\mathrm{th}}\left(\theta \right)=2^{\frac{\Psi_{e}\left(\theta \right)}{\hat{\alpha }B}}-1 \end{array}$$

## 4. Validation of the Proposed Methodology

In this section, we validated the proposed methodology considering IEEE 802.11p and the LTE-V2X sidelink. First, the proper PER value β was derived to obtain the best approximation of the PER vs. SINR curves with the step functions. Then, we estimated the best-fit implementation loss $\hat{\alpha }$ in different environments and in both LOS and NLOS conditions. Finally, based on the estimated implementation loss, we set the SINR threshold $\gamma_{\mathrm{th}}$ for the step function and used it to evaluate the performance in terms of PRR and inter-packet gap (IPG). The PRR is the percentage of the packets correctly received at a given distance, and the IPG is the time interval between two consecutive correct receptions at the same receiver from the same transmitter within a given range (set here to 150 m). The results were obtained using the open-source simulator WiLabV2Xsim [30], freely available at https://github.com/V2Xgithub/WiLabV2Xsim (accessed on 25 November 2022). The main simulation settings are listed in Table 1.

### 4.1. Derivation of the PER Value β for the Step Function Approximation

**Figure 3:**
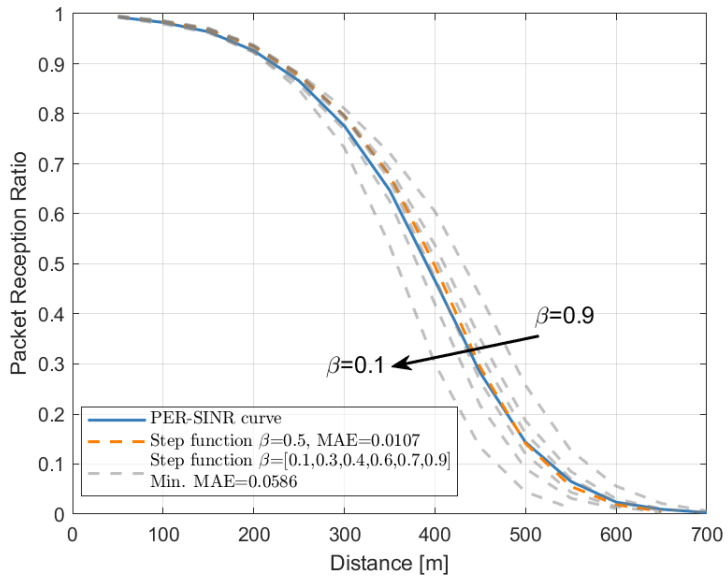
PRR vs. distance assuming the PER vs. SINR curve (solid curve) or the step function with different values of β (dashed curves). LTE-V2X MCS 7, 350 bytes, 100 vehicles/km. The orange dashed curve corresponds to β=0.5.

The suitable PER value β was derived following the approach described in Section 3.1. As an example, Figure 3 compares the communication performance in the highway LOS scenario, with 100 vehicles/km, LTE-V2X MCS 7, and a 350-byte packet size. As observable from the curves and confirmed by the minimization of the MAE, the best comparison with the solid line (i.e., the one obtained with the PER vs. SINR curve) is achieved when β=0.5, which corresponds to the orange dashed curve.

A number of additional results, assuming different technologies, MCSs, packet sizes, and vehicle densities, are also evaluated and reported in Table 2. Note that β=0.5 represents the best approximation of the PER vs. SINR model under any setting. It can also be noted that the MAE is always very small, confirming that the step function is a good approximation of the curve when looking at the network-level simulations. Given the discussed results, in the rest of the paper, β=0.5 is used.

### 4.2. Implementation Loss $\hat{\alpha }$ in the Considered Scenarios

**Figure 4:**
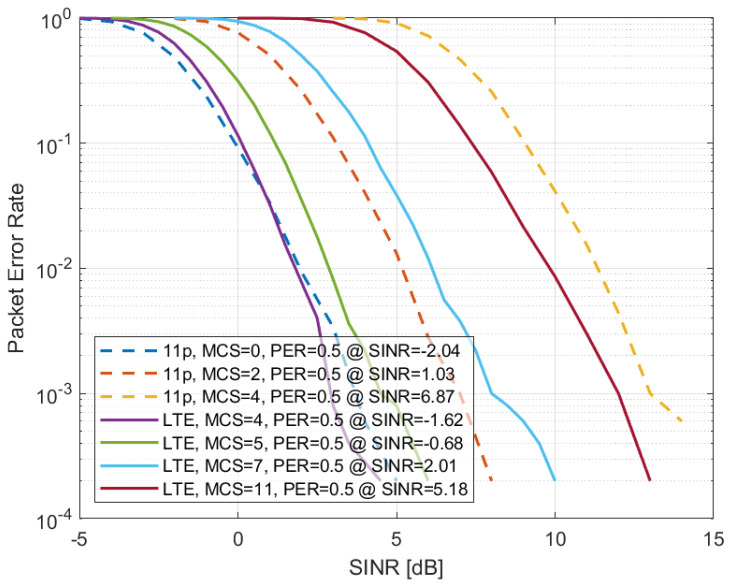
PER vs. SINR curves for IEEE 802.11p and LTE-V2X as a function of SINR in the highway LOS scenario for some of the possible MCSs and assuming packets of 350 bytes.

**Figure 5:**
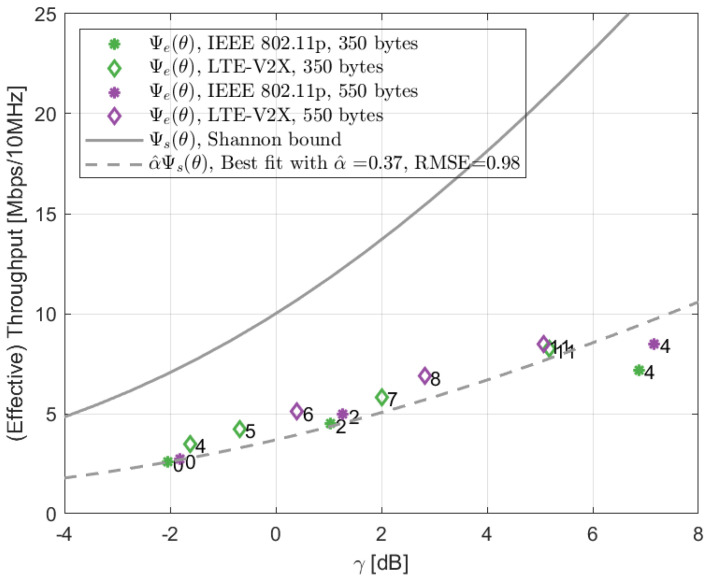
Impact of the implementation loss. The colored symbols show the effective throughput $\Psi_{e}\left(\theta \right)$ vs. SINR threshold $\gamma_{\mathrm{th}}\left(\theta \right)$ for the system settings for which the PER vs. SINR curve is available, with the numbers next to them representing the MCS indexes. The solid curve is the Shannon bound corresponding to the SINR value. The dashed line is the best fit curve with the implementation loss $\hat{\alpha }$.

Based on the dataset $\left\{\theta_{i}|i=1,2,\ldots ,N\right\}$ of measured PER vs. SINR curves (part of them are presented in Figure 4) for both the IEEE 802.11p and LTE-V2X technologies, $\gamma_{\mathrm{th}}\left(\theta_{i}\right)$ were obtained with β=0.5. Then, the estimated $\hat{\alpha }$ was derived from (10). Figure 5 represents the result of this operation for the highway LOS scenario by showing the effective throughput when varying the SINR threshold. In particular, the continuous curve corresponds to the Shannon bound. Then, each symbol indicates the effective throughput and the corresponding SINR for one of the settings for which the PER vs. SINR is available. The packet size is indicated by the color, the technology by the symbol shape, and the MCS index by the number written near the symbol. The dashed curve shows the curve obtained using the optimized implementation loss, which is, in this case, equal to 0.37. The figure confirms that the model resulting from the proposed methodology with the estimated $\hat{\alpha }$ approximates multiple system settings well. The results corresponding to other scenarios are reported in Table 3.

### 4.3. Validating Network Level Results

We now assess the effectiveness of the proposed PHY layer abstraction by evaluating the V2X communication performance both adopting the PER vs. SINR curves (as in Figure 4) and the model deriving from the proposed methodology (with the threshold SINR ${\hat{\gamma }}_{\mathrm{th}}$ obtained through (11)). Please note that the settings adopted are necessarily among those for which the curve is available, whereas the proposed methodology would allow us to also consider the other settings.

**Figure 6:**
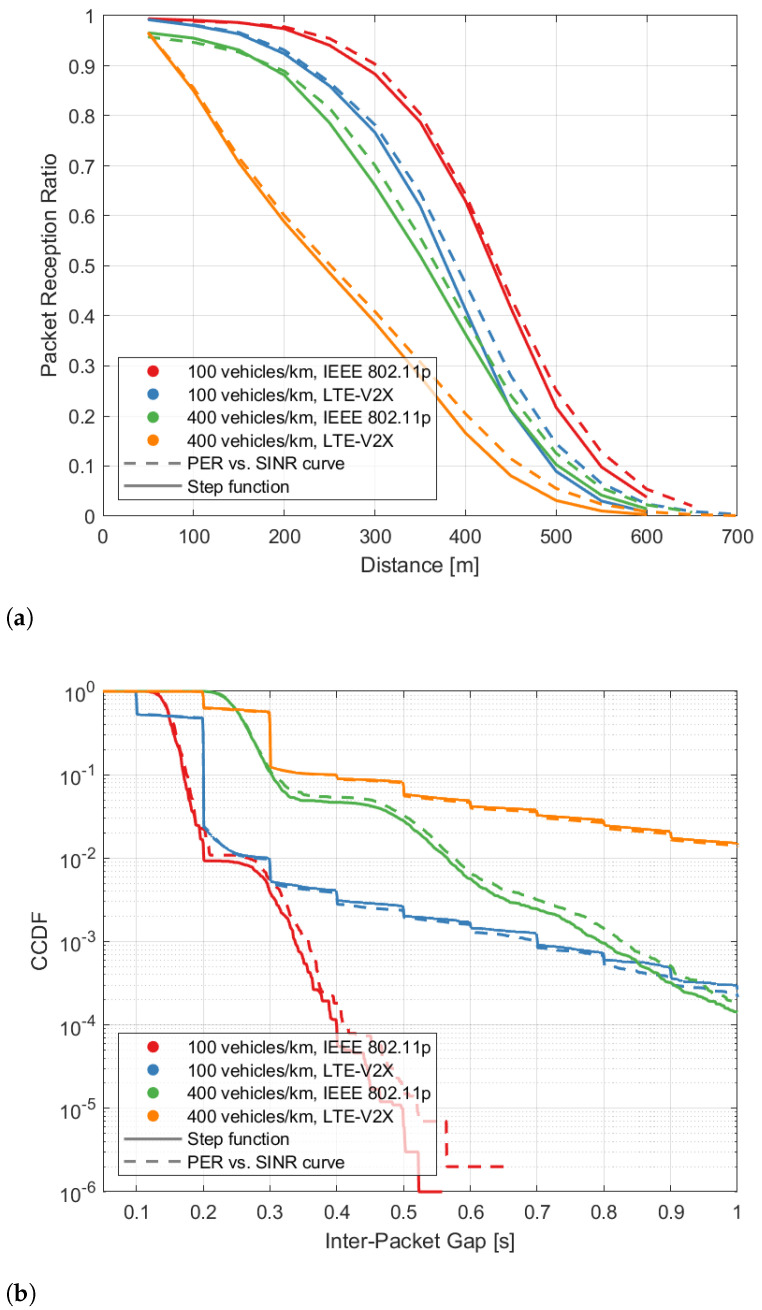
PRR vs. distance and CCDF of IPG obtained using the proposed step function approximation (solid curves) and the PER vs. SINR curves of Figure 4 (dashed curves). Results are obtained in a highway scenario with LOS conditions. (**a**) PRR vs. distance. (**b**) CCDF of IPG.

Figure 6a shows the PRR when varying the transmission distance and with a density of 100 or 400 vehicles/km. As illustrated in the figure, the results based on the proposed methodology are very close to those with the PER vs. SINR curve. The difference between the evaluated performance increases slightly for larger values of the transmission distance. Similar results shown in Figure 6b, which plots the complementary cumulative distribution function (CCDF) of the IPG, demonstrate that the proposed methodology can also evaluate IPG with a high accuracy. Overall, the slightly increased error, in terms of PRR when the distance gets larger (in Figure 6a), or in terms of IPG when a longer value is observed (in Figure 6b), appears to be negligible.

Please remark that, when adopting the proposed methodology, curves similar to those in Figure 6 can be easily obtained for both IEEE 802.11p and the LTE-V2X sidelink, in any scenario of Table 3, for any packet size, and for any MCS. Differently, if the more accurate reference model is used, new curves derived from additional measurements or link-level simulations are required for most of the possible configurations.

## 5. Conclusions

In this paper, a new methodology was proposed for modeling the PHY layer in the network-level evaluation of direct V2X communications technologies, starting from a set of PER vs. SINR curves that were first approximated with step functions and then elaborated to cover a wide set of possible configurations. The proposed methodology is general and is a low-complexity, accurate alternative to the direct use of PER vs. SINR curves, which are normally only available for a few configurations. The resulting model is characterized by a single parameter, called implementation loss, calculated here for various scenarios, and provides an accurate assessment of V2X communications without requiring costly measurement campaigns or computationally intensive simulations at the link level. The proposed approach was validated based on network-level simulations with both IEEE 802.11p and LTE-V2X sidelink technologies, while benchmarking results were obtained using the PER vs. SINR curves.

In more detail, the main findings of the work are: (i) it was shown that, in the investigated scenarios the PER vs. SINR curves can be approximated, providing a good trade-off between accuracy and complexity, with step functions obtained by using an SINR threshold corresponding to the value in the SINR vs. PER curve, where the PER is 0.5; (ii) it was shown that the curve derived from the Shannon–Hartley theorem and the addition of the implementation loss can be used to relate the configurations to the corresponding SINR thresholds; (iii) the outputs of network-level simulations performed with the proposed approach were shown to be very close to those obtained using the PER vs. SINR curves, both when looking at the error rate and the correlation among errors; (iv) the implementation loss deriving from the proposed approach and corresponding to different scenarios was provided.

In future works, the plan is to adapt and validate the model to new technologies, including IEEE 802.11bd and the NR-V2X sidelink. Nevertheless, it is worth noting that the proposed approach demonstrated a high generality when used for IEEE 802.11p and the LTE-V2X sidelink, despite their strong differences in the PHY and MAC protocols, and it is therefore likely that the same model can also be applied to other technologies.

## Figures and Tables

**Figure 1:**
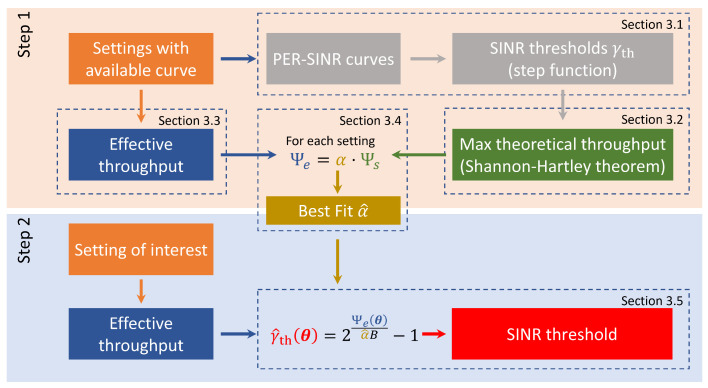
Illustration of the two main steps defining the proposed methodology for a given scenario. The first step is deriving a best fit $\hat{\alpha }$ to approximate the effective throughput based on the PER vs. SINR curves that are available (Section 3.4). The second step is to derive the SINR threshold for the settings of interest using the calculated effective throughput and the derived $\hat{\alpha }$.

**Figure 2:**
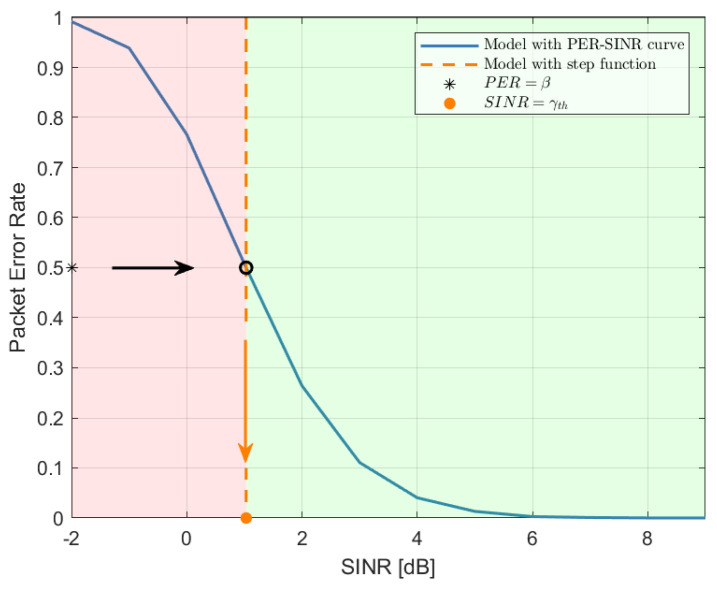
From the PER vs. SINR curve (solid blue) to the approximating step function (dashed orange). The asterisk is the target PER value β and the orange point indicates the corresponding SINR threshold $\gamma_{th}$. Adopting the step function, the packet is assumed as successfully received if the SINR is higher than the threshold (light green part), and not received otherwise (light red part).

**Table 1:** Main simulation parameters and settings.

**Scenario**	
Road layout	Highway, 3 + 3 or 6 + 6 lanes, 4 m width
(Density, Average speed) [vehicles/km, km/h]	(100, 96) and (400, 56)
**Power and propagation**	
Channels and bandwidth	ITS 10 MHz bands at 5.9 GHz
Transmission power density	13 dBm/MHz
Antenna gain (tx and rx) and noise figure	3 dBi and 6 dB
Propagation model	WINNER+, Scenario B1
Shadowing	Variance 3 dB, decorr. dist. 25 m
**Data traffic**	
Packet size and generation rule	$P_{b}=350$ bytes and following the rules in [31]
**IEEE 802.11p settings**	
MCS	2 (QPSK, CR =0.5)
Maximum contention window	15
Arbitration inter-frame space	110 μs
Sensing threshold for known and unknown signals	−85 dBm and −65 dBm
**Sidelink LTE-V2X settings**	
MCS	7 (QPSK, CR ≈0.5)
Number and size of subchannels $n_{\mathrm{subch}}$ and $n_{\mathrm{PRB}\text{-}\mathrm{subch}}$	5 and 10 PRBs
Control channel configuration	Adjacent
Retransmissions	Disabled
Keep probability	0.5
Min. and Max. time for the allocation, $T_{1}$ and $T_{2}$	1 ms and 100 ms

**Table 2:** Mean absolute error between the performance in terms of PRR vs. distance when comparing the use of the PER vs. SINR curve and the step function.

β	IEEE 802.11p				LTE-V2X			
	MCS 2, 350 Bytes		MCS 4, 550 Bytes		MCS 7, 350 Bytes		MCS 11, 550 Bytes	
	100 v/km	400 v/km	100 v/km	400 v/km	100 v/km	400 v/km	100 v/km	400 v/km
0.1	0.0621	0.0430	0.0812	0.0702	0.0586	0.0442	0.0668	0.0397
0.3	0.0184	0.0157	0.0278	0.0254	0.0215	0.0166	0.0237	0.0130
0.4	0.0086	0.0055	0.0149	0.0130	0.0125	0.0066	0.0144	0.0058
0.5	**0.0079 **	**0.0039**	**0.0106**	**0.0073**	**0.0107**	**0.0028**	**0.0077**	**0.0050**
0.6	0.0184	0.0147	0.0158	0.0112	0.0129	0.0047	0.0133	0.0099
0.7	0.0307	0.0194	0.0275	0.0230	0.0212	0.0115	0.0227	0.0189
0.9	0.0372	0.0257	0.0691	0.0521	0.0470	0.0296	0.0289	0.0334

**Table 3:** Implementation loss $\hat{\alpha }$ in different scenarios, considering different configurations $\left\{\theta_{i}|i=1,2,\ldots ,N\right\}$, varying the MCS index and packet size. The RMSE of the effective throughput with respect to the approximation is reported.

Scenarios	*N*	MCS (802.11p) and (LTE)	$P_{b}$ [bytes]	$\hat{\alpha }$	RMSE [Mb/s]
Crossing NLOS	7	(0, 2, 4) and (4,5,7,11)	350	0.25	0.82
Highway LOS	13	(0, 2, 4) and (4 8,11)	350, 550	0.37	0.98
Highway NLOS	13	(0, 2, 4) and (4 8, 11)	350, 550	0.24	0.80
Urban LOS	7	(0, 2, 4) and (4,5,7,11)	350, 550	0.32	0.99

## Data Availability

Not applicable.
